# Supplementary material for: Hybrid Nano Flake-like Vanadium Diselenide Combined on Multi-Walled Carbon Nanotube as a Binder-Free Electrode for Sodium-Ion Batteries
Source: Materials (Basel). 2023 Feb 1;16(3):1253. doi: 10.3390/ma16031253 (PMC9920653; doi:10.3390/ma16031253)
Supplement: Supplementary file 1 [file materials-16-01253-s001.zip › materials-2152636-supplementary.pdf]

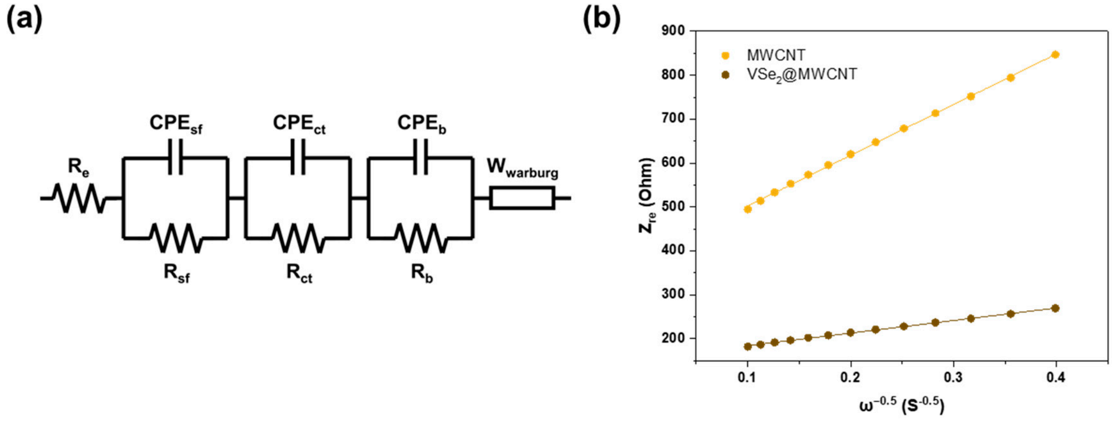

Figure S1. (**a**) equivalent circuit diagram and (b) relationship of imaginary resistance (Z′) and inverse square root of angular speed ($\omega^{-0.5}$).

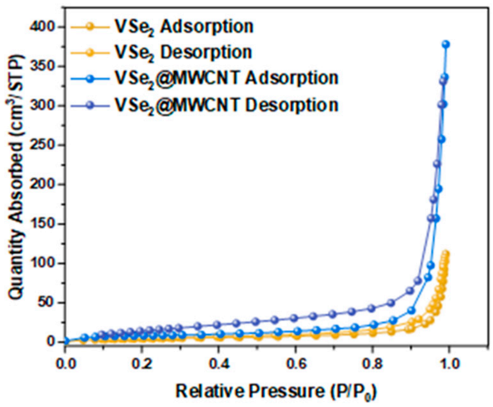

Figure S2. N_2 adsorption-desorption isotherms of VSe_2 and VSe_2@MWCNT.
